# The complete chloroplast genome of *Stephania tetrandra* (Menispermaceae)

**DOI:** 10.1080/23802359.2020.1840935

**Published:** 2020-12-24

**Authors:** Lan Cao, Zejing Mu, Shasha Sheng, Yuanyuan Chen, Guoyue Zhong, Xiaolang Du

**Affiliations:** Jiangxi University of Traditional Chinese Medicine, Nanchang, PR China

**Keywords:** Complete chloroplast genome, *Stephania tetrandra*, Menispermaceae

## Abstract

The complete chloroplast genome of *Stephania tetrandra* was sequenced and assembled for the first time. The chloroplast genome is 159,974 bp in length, containing a large single-copy (LSC) region of 90,539 bp and a small single-copy region (SSC) of 20,735 bp, separated by a pair of inverted repeats (IRs) of 24,350 bp. The genome contains 113 unique genes, including 79 protein-coding genes (PCGs), 30 *tRNA* genes, and four *rRNA* genes. Among them, 15 genes have one intron each and three genes contain two introns. The overall GC content is 37.8%, while the corresponding values of LSC, SSC, and IR regions are 35.8, 32.4, and 43.7%, respectively. Phylogenetic analysis showed that *S. tetrandra* is more closely related to the clade of two species within *Stephania*, providing new insight into the evolution of Menispermaceae.

The genus *Stephania* belongs to Menispermaceae, which has about 60 species in the world. This genus mainly occurs in the tropical and subtropical regions of Asia and Africa, with a few species are found in Oceania (Luo et al. 2008). Thirty-seven species of *Stephania* have been recorded from China (Luo et al. 2008). The plants of this genus are rich in alkaloids and are often used as pharmaceutical ingredients (Semwal et al. 2010). In this study, we sequenced and assembled the chloroplast genome of *S. tetrandra* for the first time. It will facilitate a deeper understanding and exploitation of this group.

Fresh leaves of *S. tetrandra* were collected from Anfu, Jiangxi, China (GPS: 27°17′57.328″, 114°18′43.902″). Herbarium voucher (Voucher No. JZ2020050201) is deposited in Medicinal Herbarium, Jiangxi University of Traditional Chinese Medicine, Nanchang, China. Total genomic DNA was extracted using the modified CTAB method (Doyle and Doyle 1987) and sequenced on an Illumina NovaSeq platform with paired-end reads of 150 bp. The GetOrganelle pipeline (Jin et al. 2020) was performed for the de novo assembly of complete chloroplast genome. Genes were annotated using PGA (Qu et al. 2019) and visually checked in Geneious version 8.0.2 (Kearse et al. 2012) using chloroplast genome of *S. kwangsiensis* (GenBank accession NC_048524) as a reference. The predicted transfer RNAs (tRNAs) were confirmed by tRNAscan-SE 2.0 (Lowe and Chan 2016). Finally, the complete chloroplast genome with annotations was submitted to GenBank (accession MT859132). Raw reads were deposited in the GenBank Sequence Read Archive (SRA SRR12717620).

The size of complete chloroplast genome of *S. tetrandra* is 159,974 bp with high coverage (mean 1484 ×). It has a typical quadripartite structure, including a large single-copy (LSC) region of 90,539 bp, a small single-copy region (SSC) of 20,735 bp, and a pair of inverted repeats (IRs) of 24,350 bp. There are 79 protein-coding genes (PCGs), 30 *tRNA* genes, and four *rRNA* genes. Among these genes, 15 of them (*atpF*, *ndhA*, *ndhB*, *petB*, *petD*, *rpl2*, *rpl16*, *rpoC1*, *rps16*, *trnA-UGC*, *trnG-UCC*, *trnI-GAU*, *trnK-UUU*, *trnL-UAA*, and *trnV-UAC*) are single-intron genes, and three genes (*clpP*, *rps12*, and *ycf3*) contain two introns. The overall GC content is 37.8%, while the GC content of LSC, SSC, and IR regions are 35.8, 32.4, and 43.7%, respectively.

To identify the phylogenetic relationship of *S. tetrandra* in Menispermaceae, the phylogenetic tree including *S. tetrandra*, two other species of *Stephania*, four other genera of Menispermaceae, and three outgroups (*Gymnospermium microrrhynchum*, *Sinopodophyllum hexandrum*, and *Epimedium acuminatum*) of Berberidaceae were reconstructed using complete chloroplast genomes. The sequences were aligned using MAFFT version 7.017 plugin (Katoh et al. 2002) and visually checked in Geneious. Phylogenetic analysis was performed by RAxML version 8.2 (Stamatakis 2014) using 1000 replicates of a rapid bootstrap analysis with GTRGAMMA substitution model. The phylogenetic relationships among all sampled species were fully resolved with maximum support, showing *S. tetrandra* is more closely related to the clade of two species within *Stephania*, and *Stephania* is grouped with *Pericampylus* (Figure 1). The chloroplast genome obtained in this study could provide essential data to determine the evolutionary relationship of Menispermaceae.

**Figure 1.**
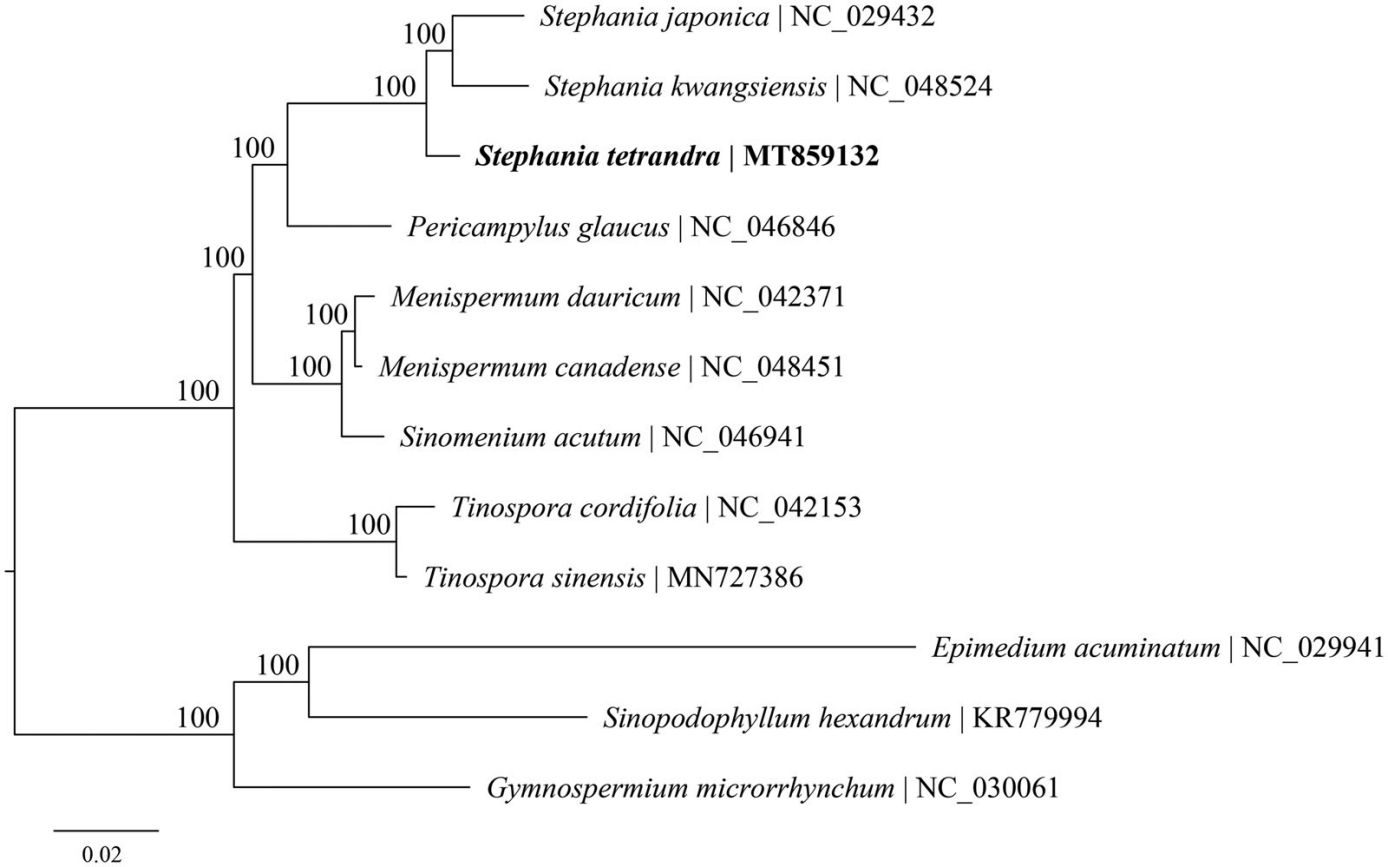
Maximum-likelihood phylogenetic tree based on complete cp genomes. Numbers close to each node are bootstrap support values.

## Data Availability

The data that support the findings of this study are openly available in GenBank at https://www.ncbi.nlm.nih.gov/genbank/, accession numbers [KR779994, NC_029941, NC_030061, MN727386, NC_029432, NC_042153, NC_042371, NC_046846, NC_046941, NC_048451, and NC_048524]. The complete chloroplast genome generated for this study has been deposited in GenBank with accession number MT859132. All high-throughput sequencing data files are available from the GenBank Sequence Read Archive (SRA) accession number: SRR12717620.
